# Transmission potential, skin inflammatory response, and parasitism of symptomatic and asymptomatic dogs with visceral leishmaniasis

**DOI:** 10.1186/1746-6148-4-45

**Published:** 2008-11-06

**Authors:** BLA Verçosa, CM Lemos, IL Mendonça, SMMS Silva, SM de Carvalho, H Goto, FAL Costa

**Affiliations:** 1 Departamento de Clinica e Cirurgia Veterinária, Centro de Ciências Agrárias, Universidade Federal do Piauí, Teresina-Pi, Brasil; 2 Departamento de Medicina Preventiva, Faculdade de Medicina, Instituto de Medicina Tropical de São Paulo, Universidade de São Paulo, São Paulo-SP, Brasil

## Abstract

**Background:**

Visceral leishmaniasis in Brazil is caused by the protozoan *Leishmania (Leishmania) chagasi *and it is transmitted by sandfly of the genus *Lutzomyia*. Dogs are an important domestic reservoir, and control of the transmission of visceral leishmaniasis (VL) to humans includes the elimination of infected dogs. However, though dogs are considered to be an important element in the transmission cycle of *Leishmania*, the identification of infected dogs representing an immediate risk for transmission has not been properly evaluated. Since it is not possible to treat infected dogs, they are sacrificed when a diagnosis of VL is established, a measure that is difficult to accomplish in highly endemic areas. In such areas, parameters that allow for easy identification of reservoirs that represents an immediate risk for transmission is of great importance for the control of VL transmission. In this study we aimed to identify clinical parameters, reinforced by pathological parameters that characterize dogs with potential to transmit the parasite to the vector.

**Results:**

The major clinical manifestations of visceral leishmaniasis in dogs from an endemic area were onicogriphosis, skin lesions, conjunctivitis, lymphadenopathy, and weight loss. The transmission potential of these dogs was assessed by xenodiagnosis using *Lutzomyia longipalpis*. Six of nine symptomatic dogs were infective to *Lutzomyia longipalpis *while none of the five asymptomatic dogs were infective to the sandfly. *Leishmania *amastigotes were present in the skin of all clinically symptomatic dogs, but absent in asymptomatic dogs. Higher parasite loads were observed in the ear and ungueal region, and lower in abdomen. The inflammatory infiltrate was more intense in the ears and ungueal regions of both symptomatic and asymptomatic dogs. In clinically affected dogs in which few or none *Leishmania *amastigotes were observed, the inflammatory infiltrate was constituted mainly of lymphocytes and macrophages. When many parasites were present, the infiltrate was also comprised of lymphocytes and macrophages, as well as a larger quantity of polymorphonuclear neutrophils (PMNs).

**Conclusion:**

Dogs that represent an immediate risk for transmission of *Leishmania *in endemic areas present clinical manifestations that include onicogriphosis, skin lesions, conjunctivitis, lymphadenopathy, and weight loss. Lymphadenopathy in particular was a positive clinical hallmark since it was closely related to the positive xenodiagnosis.

## Background

Visceral leishmaniasis (VL) in Brazil is caused by *Leishmania (Leishmania) chagasi *and it is transmitted by the sandfly *Lutzomyia longipalpis *[1]. The dog is considered to be the main domestic reservoir of *Leishmania chagasi *because it presents intense parasitism in the skin, allowing for easy transmission of *Leishmania *to the sandfly [2-4]. Therefore, dogs have been the target of control measures for the transmission of *Leishmania *to humans. In this context, the identification of infected dogs that represent an immediate risk for transmission is of utmost importance, a point that deserves meticulous study. In this study we analyzed clinical manifestation as a parameter that may indicate the immediate risk for transmission.

In endemic areas, although 67% to 80% of the animals have contact with the parasite as demonstrated either by the presence of anti-*Leishmania *antibodies or by specific cell-mediated immune response or by detection of *Leishmania*-related polymerase chain reaction products, many have no signs of disease [5-7]. In addition, symptoms suggestive of other diseases are also observed in some dogs, blurring the diagnosis of VL [8,9]. Since it is not possible to treat infected dogs, they are all sacrificed when the diagnosis of VL is established, a measure that is difficult to accomplish mainly in highly endemic areas. It is known that only some infected dogs effectively transmit the disease, that skin parasitism of dogs does not occur at the same intensity in all phases of the infection, and seemingly it does not correlate to the transmission potential to vectors [4,10,11]. Concerning transmission potential of symptomatic and asymptomatic dogs, data in the literature are controversial may be due to *Leishmania *species and geographic differences. A study carried out in Spain has shown no correlation whilst those in Colombia and Brazil have shown a positive correlation of the presence of symptoms to the infectivity [4,12,13]. In experimental *L. chagasi or L. donovani*-infected dogs infection of the vector was more likely to occur when fed on dogs at more advanced stage of the disease [14]. Therefore, the present study was aimed at identifying clinical parameters, reinforced by pathological parameters, that characterize dogs with potential to transmit the parasite to the vector in an endemic area in Brazil. We examined clinical presentations, the skin inflammatory process, parasite load and transmission potential by xenodiagnosis in dogs with visceral leishmaniasis.

## Methods

### Animals and VL diagnosis

Twenty eight dogs of this study included both privately owned and stray dogs of the endemic area of Teresina, State of Piaui, in Brazil. Male and female adult dogs of different ages and breeds were randomly tested for leishmaniasis by serology (mandatory in areas endemic for leishmaniasis) in epidemiological survey performed by the Center for Zoonosis Control. The diagnosis of VL was confirmed by a positive anti-*Leishmania *serology combined with the detection of the parasite. For the detection of anti-*Leishmania *antibodies in the sera, an indirect immunofluorescence assay or enzyme-linked immunosorbent assay were used, and for the detection of *Leishmania *we have examined directly the smears of the skin, spleen and popliteal lymph nodes stained with Giemsa, or we have performed culture of material from sternal bone marrow, spleen, and/or popliteal lymph nodes (or all) in NNN medium (SIGMA-ALDRICH). The animals were classified in three groups: a) 12 infected dogs and with clinical signs of disease; b) 11 infected dogs but without any clinical signs of VL; and c) five dogs serologically and parasitologically negative for VL as control group. Dogs were considered symptomatic when at least one of the following symptoms was present: onicogriphosis, skin lesions, loss of weight, local or generalized lymphadenopathy, diarrhea, epistaxis, conjunctivitis, anorexia, or fever. Asymptomatic dogs were those infected but without any symptoms of VL, and the diagnosis established by serological test and positive parasitological exam. The parameters used to classify clinically affected and non-affected dogs were based on the classification proposed by Pozio et al. [15].

Dogs with anemia were identified by to observe the mucosae color during necropsy. The ocular, oral, anal and/or preputial and vaginal mucosa were seen in infected dogs for the intensity of the color, compared with non-infected dogs. The pale mucosae was considered as anemia taking care to discard the possibility of hypostasis cadaverous.

### Xenodiagnosis

In nine symptomatic and five asymptomatic dogs xenodiagnosis was performed before their sacrifice for tissue sample harvest. Briefly, the dogs were anaesthetized with 1% acepromazine (0,25 mg/kg; Acepran, Univet) and 60 female *Lutzomyia longipalpis *sandflies were allowed to feed for 45 minutes on the skin of the ear. Five days after feeding, the sandflies were dissected and the middle gut removed to observe the presence of promastigotes.

### Cytological and histopathologic analysis of the skin

The animals were sedated with 1% acepromazine (0.01 ml/kg), induced with xylazin (2 mg/kg; Rompum, Bayer) and anesthetized with sodium 2.5% thiopental (0.5 ml/kg). Samples of skin were taken bilaterally from the following places: muzzle, eyelid, ear, metacarpi, forelimb ungueal region, dorsum, hind limb ungueal region, metatarsi, tail, abdomen, and scrotum. Their imprints were stained with Giemsa, and tissue samples were formalin fixed and paraffin embedded for histopathological and immunohistochemical studies. After harvest, the animals were killed with an overdose of sodium thiopental. All *Leishmania*-infected dogs were routinely sacrificed by the Center of Zoonosis for Control of VL transmission. The non-infected control animals were stray dogs from the same area that were captured and sacrificed for rabies control. All procedures involving animals were performed according to the Brazilian guide for care and use of laboratory animals (Projeto de lei 3.964/97 – ), and all experimental protocols used were previously approved by the Ethics Committee of the Federal University of Piaui.

Skin histopathological alterations were examined in all samples from 22 different locations, and the intensity was semi-quantified blindly by two independent observers, scored from 0 to 4, and the median score assessed when considering all samples from all 22 different places was used for comparison.

### Evaluation of parasite load

The parasite load was determined in cytological imprints stained with Giemsa and tissue sections stained by immunoperoxidase from all 22 different places from each animal in 50 × 100 field areas using a reticule of 10 mm^2^. Detection of *Leishmania *antigen by immunohistochemistry was performed as previously described using mouse polyclonal anti-*Leishmania (Leishmania) amazonensis *antibody that was produced by the Laboratory of Soroepidemiology and Immunobiology of Tropical Medicine Institute of University of Sao Paulo [16]. In brief, the antibody was obtained from *Leishmania (Leishmania) amazonensis *amastigote-infected BALB/c mice, tested on formalin fixed and paraffin-embedded sections of *Leishmania (L.) chagasi*-infected hamster liver (positive control) and the specificity confirmed on the same sections using serum adsorbed with *Leishmania (Leishmania) amazonensis *promastigotes when the reaction resulted negative For the reaction it was diluted 1:1.600 (vol/vol) in PBS and reacted overnight at 4°C in a humid atmosphere.

Samples were assessed using a sensitive EnVision+, peroxidase system (Dako Corporation, Carpinteria, CA, USA, Código K 4001) following protocols provided by the manufacturer.

### Statistical analyses

The data were analyzed by the Spearman's correlation or non-parametric Fisher's exact and Kruskal-Wallis tests. Those cases with a significant difference in the latter were analyzed with the Student-Newman-Keuls or Dunn test for multiple comparison of groups. We considered p < 0.05 to be significant.

## Results and discussion

All dogs except for the control group had the visceral leishmaniasis confirmed by serology and parasitological exam. Detection of dogs with VL in endemic areas is difficult because clinical signs are very variable and frequently similar to manifestations consistent with other diseases [8,9]. Our results revealed that more frequent manifestations were onicogriphosis (83.3%), skin lesions (83.3%), conjunctivitis (75%), local or generalized lymphadenopathy (66.6%), and weight loss (58.3%) (Additional file 1). Since symptomatic dogs independent of number of signs had the diagnosis of visceral leishmaniasis confirmed, we suggest these five clinical manifestations of dogs to be considered for the diagnosis of canine VL. However, in a group of 35 dogs previously studied by us two from 16 animals presenting one or two symptoms were parasitologically negative (data not shown). Therefore we suggest that only one or two of these symptoms are not sufficient, but three of them allow us to consider the animal as suspect, and five as strongly suggestive of VL. Such a clinical criteria may contribute to quickly identify dogs with visceral leishmaniasis in endemic areas.

To analyze the transmission potential of these dogs, xenodiagnosis was performed on the ear, where the parasite load was observed to be higher. From the 12 clinically affected dogs, xenodiagnosis was performed in nine. Among them, six resulted in transmission to *Lutzomyia longipalpis*, while none of the five asymptomatic dogs were infective to the sandfly. These data suggest that dogs that may represent a real threat for *Leishmania *transmission to the sandfly are those symptomatic ones (p = 0.0310, Fisher's exact test) data that are corroborated by similar results from other studies performed with *Lutzomyia longipalpis, Lutzomyia youngi *and *Phlebotomus perniciosus *[4,11]. A previous study has shown that dogs with the potential to infect the sandfly are those with at least one of the four main clinical manifestations [13]. Furthermore, five asymptomatic dogs with no parasites, as assessed by PCR of the skin in one study, and dogs with no parasites in the skin as assessed by immunohistochemistry in another study were also unable to transmit *Leishmania *to the sandfly [4,10]. On the other hand asymptomatic dogs of an endemic area in Spain have been shown to be effective to transmit *Leishmania infantum *to *Phlebotomus perniciosus *[12]. These data showing differences between studies carried out in Mediterranean region and South America may be due to *Leishmania *strain and vector species differences since it is known that *Phlebotomus perniciosus *is more effective than *Lutzomyia longipalpis *as vector to transmit viscerotropic *Leishmania *[4].

**Figure 1:**
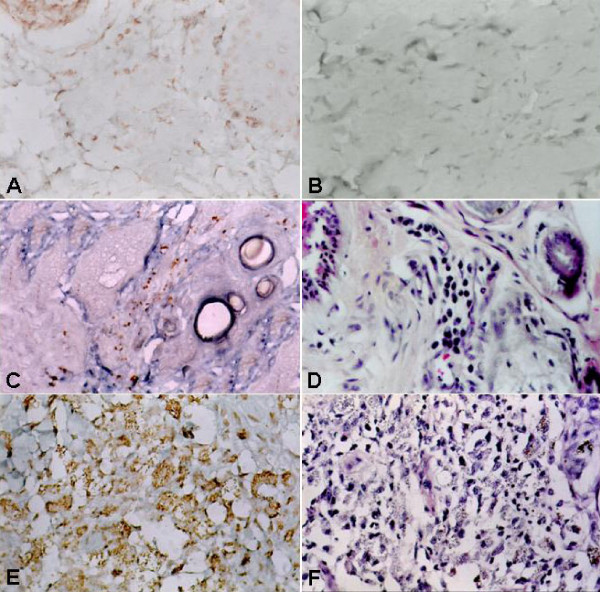
**Skin. ***Leishmania (L.) chagasi*-naturally infected (A, C, D, E, F) and non-infected dog (B). A) Absence of amastigotes in skin of the ear of asymptomatic dog; B) Absence of amastigotes in skin of the ear of control dog. C) Presence of few amastigotes in skin with minimal inflammatory infiltrate. D) Minimal inflammatory infiltrate constituted by lymphocytes, plasma cells and macrophages. E) Presence of many parasites in skin with severe inflammation. F) Severe inflammatory infiltrate constituted by macrophages and neutrophils. Imunoperoxidase staining (A, B, C, E). H-E staining (D, F). Original magnifications: ×140.

**Figure 2:**
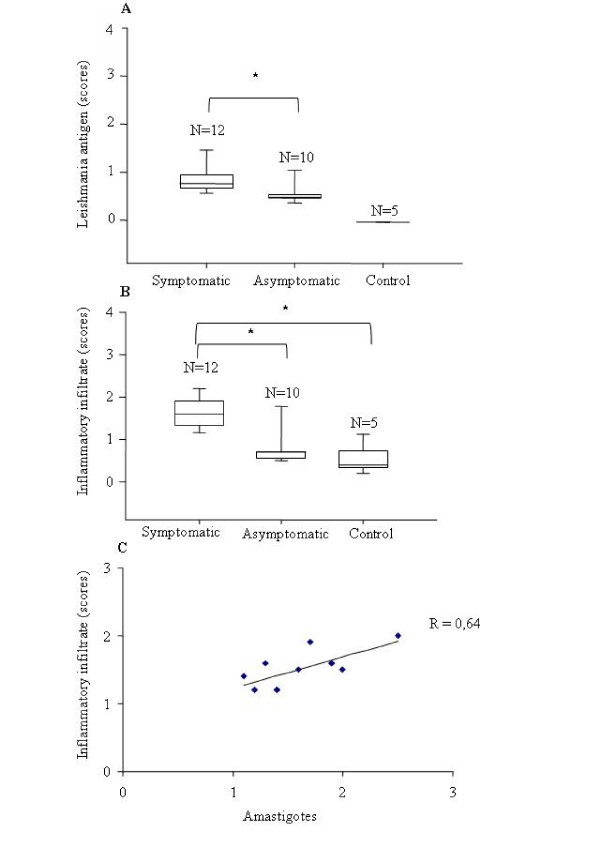
**Skin.** Dog naturally infected with *Leishmania (L.) chagasi*. A) Semi-quantitative analyses of parasite load (median scores and 25–75 percentile intervals) of clinically affected and asymptomatic and control dogs. * p < 0.05 (Kruskal Wallis and Student-Newman-Keuls tests). B) Semi-quantitative analyses of inflammatory infiltrate in the skin (median scores and 25–75 percentile intervals) in symptomatic, asymptomatic and non-infected control dogs. * p < 0.05 (Kruskal Wallis and Dunn tests). C) Correlation between presence of amastigotes and inflammatory infiltrate in symptomatic dogs. P < 0.05 (Spearman's test). N = number of animals per group.

The parasite burden in the skin was analyzed from 22 different regions of the body of dogs with VL. Higher parasite load was observed in the ear and ungueal region, and lower parasite load was observed in the abdomen using material stained by different methods. The median value is presented in Additional file 2 in tissue sections stained by immunoperoxidase. Clinically affected dogs exhibited amastigotes in at least one of the regions examined, and positive regions were always associated with inflammatory processes. In the asymptomatic (Figure 1A) and control dogs (Figure 1B), no amastigotes were present in any of the regions examined. The parasite load was higher in clinically affected dogs when compared with the asymptomatic animals (P < 0.05, Kruskal Wallis and Student-Newman-Keuls tests) (Figure 2A).

From 12 symptomatic dogs, skin lesions were observed in 10 animals. However, an inflammatory infiltrate in the skin was present in all animals, even in those without symptoms, and was more intense in the clinically affected dogs than in asymptomatic and control dogs (p = 0.006, Kruskal Wallis and Student-Newman-Keuls tests) (Figure 2B and Additional file 1). Similar results have been previously observed in *Leishmania infantum*-infected dogs [10]. Although skin inflammation is a common finding in dogs because of sandfly bites, traumas or other parasitic infections, our data showing minimal intensity of inflammatory infiltrate in the absence of amastigotes in the skin of asymptomatic and control dogs, and a correlation between the number of amastigotes and the intensity of the inflammatory infiltrate (R = 0.64; P < 0.05, Spearman test) (Figure 2C) suggest that, in clinically affected dogs, the lesions were likely related to VL [17-19].

In clinically affected dogs, when few or none *Leishmania *amastigotes were observed (Figure 1C), the inflammatory infiltrate was constituted mainly by lymphocytes and macrophages (Figure 1D). When many parasites were present (Figure 1E), the infiltrate was also comprised of lymphocytes and macrophages, as well as a larger quantity of polymorphonuclear neutrophils (PMNs) (Figure 1F). In two of these dogs (animals 5 and 7, Additional file 2), the inflammatory pattern constituted by the monomorphic macrophage infiltrate was associated with high parasite load (Fig 1E) as observed in a study by dos Santos et al. [17]. The intense inflammatory infiltrate constituted by lymphocytes, macrophages and PMN associated with high parasite load in clinically affected dogs differs from the histopathological pattern seen in tegumentar leishmaniasis, which is illustrated by defective cell-mediated immunity in which *Leishmania*-loaded macrophages are predominant and lymphocytes are scarce [20]. In asymptomatic dogs, the inflammatory infiltrate was constituted predominantly by macrophages and lymphocytes, similar to that observed in control dogs. The characteristics of the inflammatory infiltrate in clinically affected dogs with low parasite burden and the absence of amastigotes in asymptomatic dogs suggest that these animals are immune competent and therefore able to control the infection for long time.

A granulomatous inflammatory infiltrate was also observed in at least one of the regions analyzed in ten symptomatic and four asymptomatic dogs. Since the presence of a granulomatous lesion was associated with low or no parasites in the skin in four asymptomatic dogs, it suggests protective role in the tissue. Granulomatous reaction in the control of *Leishmania *infection has been studied thoroughly in *Leishmania donovani*-infected BALB/c mouse strains and also in *L. donovani*-infected hamster [21-23]. The control of the infection by granulomatous reaction depends on the cytokines secreted by inflammatory cells, mainly interferon-γ and IL 2 [24].

A parameter from the inflammatory process in the skin, the presence of PMN, seems relevant for the presence of higher parasite load. This parameter may be analyzed in smears obtained by scarification of the ear for a quick diagnostic approach.

Although the presence of *Leishmania *in the skin of the VL dog is considered to be important for the transmission of the parasite to the sandfly vector, it is not clear whether the parasites in the skin themselves are transmitted to the insect or if they are transmitted from the capillary blood to the sandfly during blood meal. If the latter is the case, the presence of parasites in the skin and the more intense inflammatory infiltrate observed are just an indication that the parasites are reaching the skin in great amounts through capillary flow. The results from the present study do not clarify this point, but suggest that the latter hypothesis is likely since, among symptomatic dogs, xenodiagnosis was positive when lymphadenopathy and/or splenomegaly were present, conditions probably related to higher parasitaemia and allowing for easy transmission to the insect. A study showing no correlation of the presence of *Leishmania *product in the skin and transmission potential to the vector also reinforces the latter hypothesis [4].

## Conclusion

The results of the present study analyzing clinical manifestations, skin inflammatory processes, parasite load in the skin and transmission potential to the sandfly vector (xenodiagnosis) clarified parameters that may identify dogs that represent an immediate risk for the transmission of *Leishmania *in endemic areas. These clinical manifestations can be detected easily, including the presence of PMNs in inflammatory processes of the skin. Five clinical signs to be considered together as strongly suggestive of VL are abnormal nails, skin lesions, conjunctivitis, lymphadenopathy, and weight loss. Lymphadenopathy, in particular, was a positive clinical hallmark since it was closely related to the positive xenodiagnosis. Targeting animals with these clinical signs to perform serological tests and parasitological exams would accelerate the identification of animals with actual potential risk for transmission, contributing to VL transmission control in endemic areas.

## Authors' contributions

BLAV participated in sample harvest, performance of assays, data analysis and manuscript preparation. HG participated in the development of the study including analysis of the data and manuscript revision. CML participated in sample harvest, performance of assays and data analysis. ILM, SMMSS and SMC participated in discussion of the project, technical support and contributed to the manuscript preparation. FALC conceived of the study and coordinated all activity of the present study, from sample harvest, performance of different assays, analysis of data and manuscript preparation. All authors read and approved of the final manuscript.

## Supplementary Material

Additional file 1 Table 1. Clinical signals, xenodiagonosis and inflammatory infiltrate in 23 *Leishmania (L.) chagasi*-naturally infected serologically and parasitologically positive dogs, Xeno = xenodiagnosis; Ly = lymphocyte; Mf = macrophage; PMN = polymorphonuclear; ND = not done Click here for file

Additional file 2 Table 2. Semi-quantitative analysis of amastigotes in skin of *Leishmania (L.) chagasi*-naturally infected dogs. Imunoperoxidase Click here for file
