# Supplementary material for: Transmission potential, skin inflammatory response, and parasitism of symptomatic and asymptomatic dogs with visceral leishmaniasis
Source: BMC Vet Res. 2008 Nov 6;4:45. doi: 10.1186/1746-6148-4-45 (PMC2613136; doi:10.1186/1746-6148-4-45)
Supplement: Additional file 1 — Table 1. Clinical signals, xenodiagonosis and inflammatory infiltrate in 23 Leishmania (L.) chagasi-naturally infected serologically and parasitologically positive dogs, Xeno = xenodiagnosis; Ly = lymphocyte; Mf = macrophage; PMN = polymorphonuclear; ND = not done [file 1746-6148-4-45-S1.doc]

| Animals | Clinical signals | | | | | | | | Xeno | Inflammatory infiltrate | | | |
| --- | --- | --- | --- | --- | --- | --- | --- | --- | --- | --- | --- | --- | --- |
|  | Fever  (41,7%) | Anaemia  (58,3%) | Weight loss  (58,3%) | Skin lesions  (83,3%) | Abnormal nails  (83,3%) | Conjunctivitis  (75%) | Adeno  pathy  (66,7%) | Splenomegaly  (50%) |  | Ly | Mf | Plasma cells | PMN |
| 1 | - | + | + | + | + | - | + | + | + | +++ | +++ | +++ | - |
| 2 | + | + | + | + | + | + | + | + | + | +++ | +++ | +++ | + |
| 3 | + | + | + | + | + | + | + | - | - | +++ | +++ | +++ | + |
| 4 | - | + | + | + | + | + | - | + | - | +++ | +++ | - | +++ |
| 5 | + | - | - | + | + | + | + | + | + | + | +++ | + | +++ |
| 6 | - | - | + | + | + | + | + | + | + | +++ | +++ | - | +++ |
| 7 | + | + | + | - | + | + | + | - | ND | - | +++ | - | +++ |
| 8 | + | - | + | - | + | + | - | - | - | - | +++ | +++ | + |
| 9 | - | + | - | + | + | + | + | - | + | +++ | +++ | +++ | + |
| 10 | - | + | - | + | + | + | + | + | + | +++ | +++ | - | + |
| 11 | - | - | - | + | - | - | - | - | ND | +++ | +++ | - | - |
| 12 | - | - | - | + | - | - | - | - | ND | +++ | +++ | - | - |
| 13 | - | - | - | - | - | - | - | - | ND | +++ | - | +++ | - |
| 14 | - | - | - | - | - | - | - | - | - | +++ | +++ | +++ | - |
| 15 | - | - | - | - | - | - | - | - | - | + | +++ | - | - |
| 16 | - | - | - | - | - | - | - | - | - | +++ | +++ | - | - |
| 17 | - | - | - | - | - | - | - | - | - | - | +++ | - | - |
| 18 | - | - | - | - | - | - | - | - | ND | +++ | +++ | - | - |
| 19 | - | - | - | - | - | - | - | - | ND | +++ | +++ | - | - |
| 20 | - | - | - | - | - | - | - | - | ND | +++ | +++ | - | - |
| 21 | - | - | - | - | - | - | - | - | ND | +++ | +++ | - | - |
| 22 | - | - | - | - | - | - | - | - | ND | +++ | +++ | - | - |
| 23 | - | - | - | - | - | - | - | - | - | - | - | - | - |

Table 1. Clinical signals, xenodiagonosis and inflammatory infiltrate in 23 *Leishmania (L.) chagasi*-naturally infected serologically and parasitologically positive

dogs, Xeno = xenodiagnosis; Ly = lymphocyte; Mf = macrophage; PMN = polymorphonuclear; ND = not done
