# Supplementary material for: Transmission potential, skin inflammatory response, and parasitism of symptomatic and asymptomatic dogs with visceral leishmaniasis
Source: BMC Vet Res. 2008 Nov 6;4:45. doi: 10.1186/1746-6148-4-45 (PMC2613136; doi:10.1186/1746-6148-4-45)
Supplement: Additional file 2 — Table 2. Semi-quantitative analysis of amastigotes in skin of Leishmania (L.) chagasi-naturally infected dogs. Imunoperoxidase [file 1746-6148-4-45-S2.doc]

| Animals | Muzzle | Eyelid | Ear | Meta  carpi | Forelimb  ungueal | Dorsum | Metatarsi | Hind limb  ungueal | Tail | Abdomen | Scrotum |
| --- | --- | --- | --- | --- | --- | --- | --- | --- | --- | --- | --- |
| 1 | 1.5 | 2 | 2.5 | 1 | 1.5 | 1.5 | 1 | 2 | 1 | 1 | 0 |
| 2 | 1 | 1 | 1 | 0 | 1 | 0.5 | 0 | 1 | 1 | 0 | 1 |
| 3 | 0.5 | 0.5 | 1.5 | 2 | 1 | 1 | 0.5 | 0.5 | 1 | 1 | 1 |
| 4 | 1 | 1 | 1.5 | 1 | 1 | 1 | 0 | 0.5 | 1 | 0 | 1 |
| 5 | 1.5 | 2.5 | 4 | 3 | 3 | 2 | 1 | 2 | 2 | 2 | 0 |
| 6 | 2 | 2 | 1.5 | 1 | 1 | 1.5 | 2 | 1.5 | 2 | 1 | 3 |
| 7 | 1 | 3 | 3.5 | 2.5 | 2.5 | 1.5 | 2 | 1.5 | 2 | 2 | 2 |
| 8 | 1.5 | 1.5 | 3 | 2 | 2 | 2 | 1.5 | 3 | 1 | 2 | 2 |
| 9 | 2.5 | 2 | 2 | 2 | 3 | 2 | 2.5 | 3 | 2 | 1 | 0 |
| 10 | 1 | 1.5 | 3 | 1 | 1.5 | 2 | 2 | 3 | 2 | 1 | 0 |
| 11 | 1 | 1.5 | 1.5 | 2 | 1.5 | 2.5 | 2.5 | 1 | 2 | 1 | 3 |
| 12 | 1 | 1 | 2 | 2 | 2 | 1 | 1 | 2 | 1 | 1 | 0 |
| **Median** | **1.3** | **1.6** | **2.2** | **1.6** | **1.8** | **1.5** | **1.3** | **1.8** | **1.5** | **1.1** | **1.1** |
